# Di-μ-chlorido-bis­{chlorido[2,3-dimethyl-*N*-(pyridin-2-yl­methyl­idene)aniline-κ^2^ *N*,*N*′]mercury(II)}

**DOI:** 10.1107/S1600536812039050

**Published:** 2012-09-22

**Authors:** Seyed Jalal Hoseyni, Mohamad Reza Talei Bavil Olyai, Behrouz Notash

**Affiliations:** a Department of Chemistry, Islamic Azad University, Karaj Branch, Karaj, Iran; b Department of Chemistry, Faculty of Science, Islamic Azad University, South Tehran Branch, Tehran, Iran; c Department of Chemistry, Shahid Beheshti University, G. C., Evin, Tehran 1983963113, Iran

## Abstract

In the centrosymmetric binuclear molecule of the title complex, [Hg_2_Cl_4_(C_14_H_14_N_2_)_2_], the five-coordinated Hg^II^ ions have a distorted square-pyramidal geometry defined by two N atoms belonging to the chelating imino­pyridine ligand and three Cl atoms. The benzene and pyridine rings are oriented at a dihedral angle of 56.7 (6)°. The crystal packing is stabilized by C—H⋯Cl hydrogen bonds and π–π inter­actions between the pyridine rings [centroid–centroid distance = 3.796 (6) Å].

## Related literature

For background to Schiff base compounds, see: Gibson *et al.* (2007 ▶); Gibson & Spitzmesser (2003 ▶); Ittel *et al.* (2000 ▶). For related structures, see: Baul *et al.* (2004 ▶).
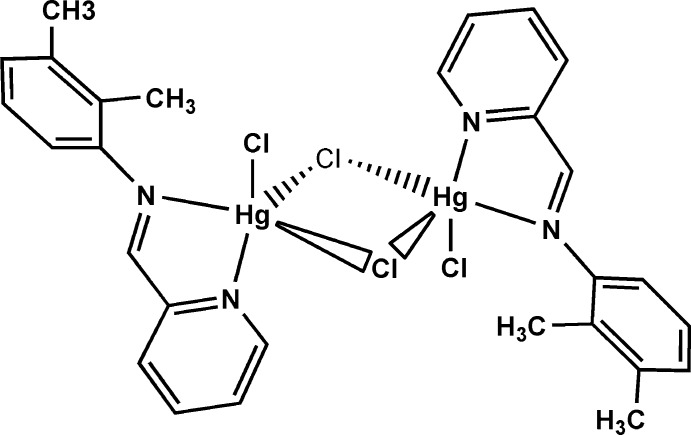


## Experimental

### 

#### Crystal data

[Hg_2_Cl_4_(C_14_H_14_N_2_)_2_]
*M* *_r_* = 963.52
Monoclinic, 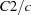

*a* = 7.7989 (16) Å
*b* = 26.525 (5) Å
*c* = 15.098 (3) Å
β = 98.26 (3)°
*V* = 3090.9 (11) Å^3^
*Z* = 4
Mo *K*α radiation
μ = 10.29 mm^−1^
*T* = 298 K
0.50 × 0.17 × 0.15 mm

#### Data collection

Stoe IPDS-2T diffractometer
Absorption correction: numerical (*X-SHAPE* and *X-RED*; Stoe & Cie, 2002 ▶) *T*_min_ = 0.126, *T*_max_ = 0.210
12121 measured reflections
4164 independent reflections
3174 reflections with *I* > 2σ(*I*)
*R*_int_ = 0.118

#### Refinement

*R*[*F* ^2^ > 2σ(*F* ^2^)] = 0.083
*wR*(*F* ^2^) = 0.206
*S* = 1.11
4164 reflections
175 parameters
H-atom parameters constrained
Δρ_max_ = 3.28 e Å^−3^
Δρ_min_ = −4.88 e Å^−3^

### 

Data collection: *X-AREA* (Stoe & Cie, 2002 ▶); cell refinement: *X-AREA*; data reduction: *X-RED* (Stoe & Cie, 2002 ▶); program(s) used to solve structure: *SHELXS97* (Sheldrick, 2008 ▶); program(s) used to refine structure: *SHELXL97* (Sheldrick, 2008 ▶); molecular graphics: *ORTEP-3* (Farrugia, 1997 ▶) and *Mercury* (Macrae *et al.*, 2006 ▶); software used to prepare material for publication: *WinGX* (Farrugia, 1999 ▶).

## Supplementary Material

Crystal structure: contains datablock(s) I, global. DOI: 10.1107/S1600536812039050/hy2585sup1.cif

Structure factors: contains datablock(s) I. DOI: 10.1107/S1600536812039050/hy2585Isup2.hkl

Additional supplementary materials:  crystallographic information; 3D view; checkCIF report

## Figures and Tables

**Table 1:** Hydrogen-bond geometry (Å, °)

*D*—H⋯*A*	*D*—H	H⋯*A*	*D*⋯*A*	*D*—H⋯*A*
C1—H1⋯Cl2^i^	0.93	2.65	3.400 (11)	138
C4—H4⋯Cl2^ii^	0.93	2.67	3.552 (11)	158
C14—H14*C*⋯Cl1^iii^	0.96	2.76	3.685 (14)	162

Symmetry codes: (i) 
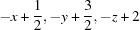
; (ii) 
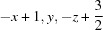
; (iii) 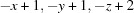.

**Fig. 1.**
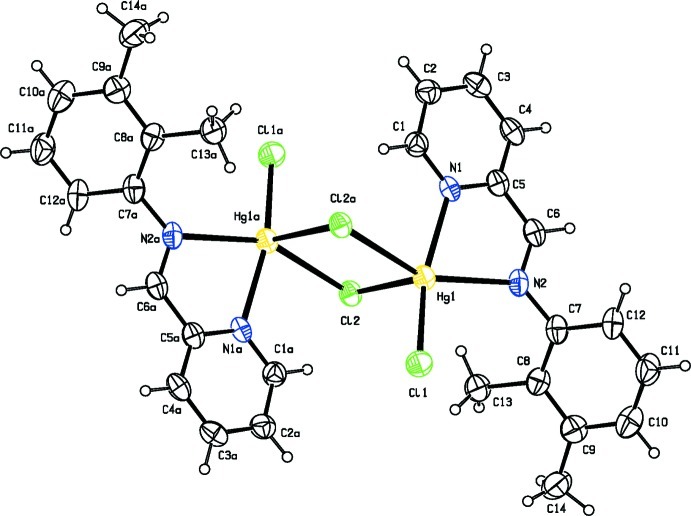
The molecular structure of the title compound. Displacement ellipsoids are drawn at the 30% probability level. [Symmetry code: (a) 1/2-x, 3/2-y, 2-z.]

**Fig. 2.**
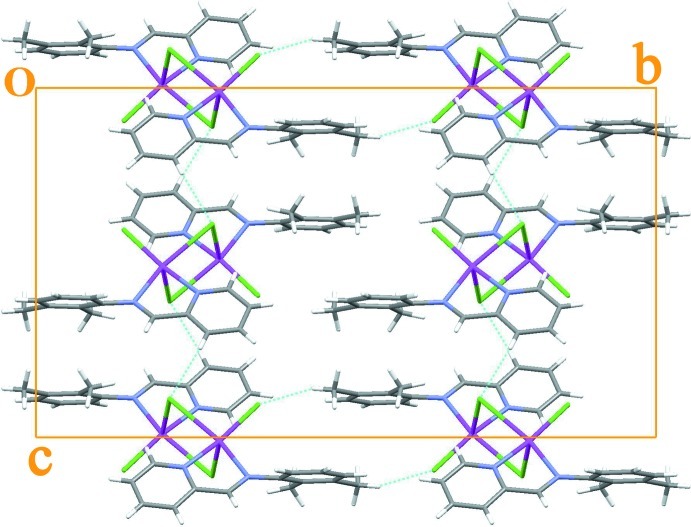
Packing diagram of the title compound. Hydrogen bonds are shown as blue dashed lines.

## supplementary crystallographic information

### Comment

Schiff base metal complexes have been known since the nineteenth century. Investigation on metal-organic complexes represents one of the most active areas of material science and chemical research (Gibson *et al.*, 2007; Gibson & Spitzmesser, 2003; Ittel *et al.*, 2000). Schiff bases form a class of compounds with azomethin group, which are usually synthesized from the condensation of primary amines and active carbonyl groups by the elimination of water molecule. We report herein the crystal structure of the title compound, a new mercury(II) complex. This complex was synthesized by the reaction of HgCl_2_ with 2-[(2,3-dimethylphenyl)iminomethyl]pyridine in an acetonitril solution.

In the title compound (Fig. 1), the Hg^II^ atom is five-coordinated in a distorted squar-pyramidal geometry. The Schiff base ligand coordinates to the Hg^II^ atom as a bidentate ligand through the N atoms of the imine group and pyridine ring. Also two bridging and one terminal chloride anions are present in the coordination environment of the Hg^II^ atom (Baul *et al.*, 2004). The benzene and pyridine rings are oriented at a dihedral angle of 56.7 (6)°. Crystal packing is stabilized by intermolecular C—H···Cl hydrogen bonds (Fig. 2, Table 1) and π–π interactions between the pyridine rings [centroid–centroid distance = 3.796 (6) Å].

### Experimental

For the preparation of the title compound, a solution of 2-[(2,3-dimethylphenyl)iminomethyl]pyridine (0.210 g, 1.00 mmol) in acetonitril (10 ml) was added slowly to a solution of HgCl_2_ (0.271 g, 1.00 mmol) in acetonitril (10 ml) and the resulting yellow solution was stirred for 45 min at room temperature. Then the yellow precipitate was filtered and dissolved in acetonitril and left to evaporate slowly at -18°C. After a few days, yellow crystals of the title compound were isolated (yield: 0.272 g, 56.5%; m.p. 489 K).

### Refinement

H atoms were positioned geometrically and refined as riding atoms, with C—H = 0.93 (CH) and 0.96 (CH_3_) Å and with *U*_iso_(H) = 1.2(1.5 for methyl)*U*_eq_(C). The highest residual electron density was found at 0.85 Å from Hg1 atom and the deepest hole at 0.92 Å from Hg1 atom.

### Crystal data

**Table:**

[Hg_2_Cl_4_(C_14_H_14_N_2_)_2_]	*F*(000) = 1808.0
*M**_r_* = 963.52	*D*_x_ = 2.071 Mg m^−^^3^
Monoclinic, *C*2/*c*	Mo *K*α radiation, λ = 0.71073 Å
Hall symbol: -C 2yc	Cell parameters from 4164 reflections
*a* = 7.7989 (16) Å	θ = 2.1–29.2°
*b* = 26.525 (5) Å	µ = 10.29 mm^−^^1^
*c* = 15.098 (3) Å	*T* = 298 K
β = 98.26 (3)°	Column, yellow
*V* = 3090.9 (11) Å^3^	0.50 × 0.17 × 0.15 mm
*Z* = 4	

### Data collection

**Table:**

Stoe IPDS-2T diffractometer	4164 independent reflections
Radiation source: fine-focus sealed tube	3174 reflections with *I* > 2σ(*I*)
Graphite monochromator	*R*_int_ = 0.118
Detector resolution: 0.15 mm pixels mm^-1^	θ_max_ = 29.2°, θ_min_ = 2.1°
ω scans	*h* = −10→10
Absorption correction: numerical (*X-SHAPE* and *X-RED*; Stoe & Cie, 2002)	*k* = −36→35
*T*_min_ = 0.126, *T*_max_ = 0.210	*l* = −20→16
12121 measured reflections	

### Refinement

**Table:**

Refinement on *F*^2^	Secondary atom site location: difference Fourier map
Least-squares matrix: full	Hydrogen site location: inferred from neighbouring sites
*R*[*F*^2^ > 2σ(*F*^2^)] = 0.083	H-atom parameters constrained
*wR*(*F*^2^) = 0.206	*w* = 1/[σ^2^(*F*_o_^2^) + (0.1219*P*)^2^] where *P* = (*F*_o_^2^ + 2*F*_c_^2^)/3
*S* = 1.11	(Δ/σ)_max_ = 0.001
4164 reflections	Δρ_max_ = 3.28 e Å^−^^3^
175 parameters	Δρ_min_ = −4.88 e Å^−^^3^
0 restraints	Extinction correction: *SHELXL97* (Sheldrick, 2008), Fc^*^=kFc[1+0.001xFc^2^λ^3^/sin(2θ)]^-1/4^
Primary atom site location: structure-invariant direct methods	Extinction coefficient: 0.0012 (2)

### Special details

**Table:**

Geometry. All e.s.d.'s (except the e.s.d. in the dihedral angle between two l.s. planes) are estimated using the full covariance matrix. The cell e.s.d.'s are taken into account individually in the estimation of e.s.d.'s in distances, angles and torsion angles; correlations between e.s.d.'s in cell parameters are only used when they are defined by crystal symmetry. An approximate (isotropic) treatment of cell e.s.d.'s is used for estimating e.s.d.'s involving l.s. planes.
Refinement. Refinement of *F*^2^ against ALL reflections. The weighted *R*-factor *wR* and goodness of fit *S* are based on *F*^2^, conventional *R*-factors *R* are based on *F*, with *F* set to zero for negative *F*^2^. The threshold expression of *F*^2^ > σ(*F*^2^) is used only for calculating *R*-factors(gt) *etc*. and is not relevant to the choice of reflections for refinement. *R*-factors based on *F*^2^ are statistically about twice as large as those based on *F*, and *R*- factors based on ALL data will be even larger.

### Fractional atomic coordinates and isotropic or equivalent isotropic displacement parameters (Å^2^)

**Table:**

	*x*	*y*	*z*	*U*_iso_*/*U*_eq_	
Hg1	0.43296 (5)	0.703848 (14)	0.98975 (3)	0.0525 (2)	
Cl2	0.1308 (3)	0.71572 (10)	0.88978 (17)	0.0524 (5)	
Cl1	0.3754 (4)	0.64201 (11)	1.0936 (2)	0.0663 (7)	
N1	0.5738 (9)	0.7612 (3)	0.9145 (5)	0.0452 (16)	
N2	0.6082 (10)	0.6579 (3)	0.8873 (6)	0.0528 (19)	
C5	0.6666 (12)	0.7441 (4)	0.8523 (6)	0.050 (2)	
C6	0.6903 (13)	0.6896 (4)	0.8436 (7)	0.052 (2)	
H6	0.7656	0.6776	0.8060	0.062*	
C8	0.5112 (14)	0.5709 (4)	0.8740 (7)	0.057 (2)	
C7	0.6450 (14)	0.6053 (4)	0.8836 (7)	0.058 (2)	
C1	0.5528 (15)	0.8102 (4)	0.9243 (7)	0.055 (2)	
H1	0.4887	0.8214	0.9678	0.066*	
C13	0.3227 (15)	0.5888 (5)	0.8577 (9)	0.065 (3)	
H13A	0.2696	0.5828	0.9102	0.098*	
H13B	0.2608	0.5706	0.8081	0.098*	
H13C	0.3194	0.6242	0.8445	0.098*	
C12	0.8192 (15)	0.5889 (5)	0.8986 (8)	0.065 (3)	
H12	0.9093	0.6122	0.9065	0.078*	
C9	0.5443 (17)	0.5190 (5)	0.8768 (9)	0.068 (3)	
C2	0.6211 (14)	0.8450 (4)	0.8733 (8)	0.060 (2)	
H2	0.6030	0.8792	0.8814	0.072*	
C4	0.7403 (13)	0.7770 (5)	0.7992 (7)	0.056 (2)	
H4	0.8051	0.7649	0.7566	0.068*	
C3	0.7178 (14)	0.8285 (5)	0.8093 (8)	0.062 (3)	
H3	0.7669	0.8515	0.7736	0.074*	
C14	0.406 (2)	0.4806 (5)	0.8639 (13)	0.092 (5)	
H14A	0.3288	0.4876	0.8098	0.138*	
H14B	0.3418	0.4814	0.9138	0.138*	
H14C	0.4561	0.4478	0.8599	0.138*	
C11	0.8530 (17)	0.5385 (5)	0.9014 (10)	0.080 (4)	
H11	0.9672	0.5273	0.9101	0.096*	
C10	0.7187 (19)	0.5035 (6)	0.8912 (10)	0.082 (4)	
H10	0.7446	0.4693	0.8940	0.098*	

### Atomic displacement parameters (Å^2^)

**Table:**

	*U*^11^	*U*^22^	*U*^33^	*U*^12^	*U*^13^	*U*^23^
Hg1	0.0468 (3)	0.0595 (3)	0.0521 (3)	0.00288 (15)	0.00989 (16)	0.00296 (15)
Cl2	0.0445 (11)	0.0637 (13)	0.0479 (11)	0.0022 (10)	0.0026 (9)	0.0021 (10)
Cl1	0.0688 (17)	0.0655 (14)	0.0649 (16)	0.0064 (12)	0.0102 (13)	0.0160 (12)
N1	0.034 (3)	0.064 (4)	0.037 (3)	−0.003 (3)	0.006 (3)	−0.009 (3)
N2	0.041 (4)	0.063 (5)	0.055 (4)	0.007 (4)	0.008 (3)	−0.013 (4)
C5	0.039 (4)	0.067 (6)	0.043 (4)	0.002 (4)	−0.002 (4)	0.000 (4)
C6	0.039 (4)	0.075 (6)	0.041 (4)	0.002 (4)	0.002 (4)	−0.006 (4)
C8	0.055 (5)	0.062 (5)	0.051 (5)	0.009 (5)	−0.003 (4)	−0.004 (4)
C7	0.047 (5)	0.073 (6)	0.053 (5)	0.009 (5)	0.003 (4)	−0.009 (5)
C1	0.056 (6)	0.058 (5)	0.053 (5)	0.004 (4)	0.012 (5)	0.003 (4)
C13	0.054 (6)	0.070 (6)	0.068 (7)	0.001 (5)	−0.002 (5)	0.001 (5)
C12	0.052 (6)	0.079 (7)	0.063 (6)	0.020 (5)	0.006 (5)	−0.010 (5)
C9	0.066 (7)	0.071 (7)	0.065 (7)	0.001 (6)	0.002 (5)	0.001 (5)
C2	0.050 (5)	0.062 (6)	0.064 (6)	−0.004 (5)	−0.001 (5)	0.004 (5)
C4	0.041 (5)	0.084 (7)	0.043 (5)	−0.006 (5)	0.004 (4)	−0.002 (5)
C3	0.052 (6)	0.079 (7)	0.053 (5)	−0.013 (5)	0.003 (5)	0.009 (5)
C14	0.089 (10)	0.059 (7)	0.125 (14)	0.008 (6)	0.001 (9)	0.001 (8)
C11	0.057 (7)	0.083 (8)	0.096 (10)	0.019 (6)	−0.005 (7)	−0.004 (7)
C10	0.083 (9)	0.074 (8)	0.084 (9)	0.022 (7)	−0.003 (7)	−0.005 (7)

### Geometric parameters (Å, º)

**Table:**

Hg1—N1	2.273 (8)	C13—H13A	0.9600
Hg1—Cl1	2.356 (3)	C13—H13B	0.9600
Hg1—N2	2.521 (8)	C13—H13C	0.9600
Hg1—Cl2	2.628 (3)	C12—C11	1.363 (18)
Hg1—Cl2^i^	2.893 (3)	C12—H12	0.9300
N1—C1	1.322 (14)	C9—C10	1.407 (18)
N1—C5	1.345 (13)	C9—C14	1.479 (19)
N2—C6	1.295 (14)	C2—C3	1.379 (17)
N2—C7	1.426 (14)	C2—H2	0.9300
C5—C4	1.365 (16)	C4—C3	1.390 (18)
C5—C6	1.466 (15)	C4—H4	0.9300
C6—H6	0.9300	C3—H3	0.9300
C8—C7	1.379 (16)	C14—H14A	0.9600
C8—C9	1.400 (17)	C14—H14B	0.9600
C8—C13	1.531 (15)	C14—H14C	0.9600
C7—C12	1.413 (14)	C11—C10	1.39 (2)
C1—C2	1.358 (16)	C11—H11	0.9300
C1—H1	0.9300	C10—H10	0.9300
			
N1—Hg1—Cl1	161.8 (2)	C8—C13—H13B	109.5
N1—Hg1—N2	70.9 (3)	H13A—C13—H13B	109.5
Cl1—Hg1—N2	104.1 (2)	C8—C13—H13C	109.5
N1—Hg1—Cl2	95.2 (2)	H13A—C13—H13C	109.5
Cl1—Hg1—Cl2	102.99 (10)	H13B—C13—H13C	109.5
N2—Hg1—Cl2	103.1 (2)	C11—C12—C7	119.0 (13)
N1—Hg1—Cl2^i^	87.7 (2)	C11—C12—H12	120.5
Cl1—Hg1—Cl2^i^	91.64 (10)	C7—C12—H12	120.5
N2—Hg1—Cl2^i^	154.2 (2)	C8—C9—C10	117.5 (12)
Cl2—Hg1—Cl2^i^	92.97 (7)	C8—C9—C14	123.0 (11)
Hg1—Cl2—Hg1^i^	87.03 (7)	C10—C9—C14	119.4 (13)
C1—N1—C5	119.7 (9)	C1—C2—C3	118.8 (11)
C1—N1—Hg1	121.8 (7)	C1—C2—H2	120.6
C5—N1—Hg1	118.2 (7)	C3—C2—H2	120.6
C6—N2—C7	119.9 (9)	C5—C4—C3	119.6 (10)
C6—N2—Hg1	110.4 (7)	C5—C4—H4	120.2
C7—N2—Hg1	128.9 (7)	C3—C4—H4	120.2
N1—C5—C4	120.7 (10)	C2—C3—C4	118.5 (10)
N1—C5—C6	118.7 (9)	C2—C3—H3	120.7
C4—C5—C6	120.6 (10)	C4—C3—H3	120.7
N2—C6—C5	121.2 (9)	C9—C14—H14A	109.5
N2—C6—H6	119.4	C9—C14—H14B	109.5
C5—C6—H6	119.4	H14A—C14—H14B	109.5
C7—C8—C9	121.0 (11)	C9—C14—H14C	109.5
C7—C8—C13	120.4 (10)	H14A—C14—H14C	109.5
C9—C8—C13	118.6 (11)	H14B—C14—H14C	109.5
C8—C7—C12	120.6 (11)	C12—C11—C10	120.8 (12)
C8—C7—N2	119.9 (9)	C12—C11—H11	119.6
C12—C7—N2	119.3 (10)	C10—C11—H11	119.6
N1—C1—C2	122.7 (11)	C11—C10—C9	121.2 (12)
N1—C1—H1	118.6	C11—C10—H10	119.4
C2—C1—H1	118.6	C9—C10—H10	119.4
C8—C13—H13A	109.5		
			
N1—Hg1—Cl2—Hg1^i^	−88.0 (2)	C4—C5—C6—N2	−173.4 (9)
Cl1—Hg1—Cl2—Hg1^i^	92.38 (10)	C9—C8—C7—C12	−1.6 (18)
N2—Hg1—Cl2—Hg1^i^	−159.6 (2)	C13—C8—C7—C12	−180.0 (11)
Cl2^i^—Hg1—Cl2—Hg1^i^	0.0	C9—C8—C7—N2	−175.2 (11)
Cl1—Hg1—N1—C1	−105.9 (9)	C13—C8—C7—N2	6.4 (16)
N2—Hg1—N1—C1	177.2 (8)	C6—N2—C7—C8	−137.2 (11)
Cl2—Hg1—N1—C1	75.2 (7)	Hg1—N2—C7—C8	54.0 (13)
Cl2^i^—Hg1—N1—C1	−17.6 (7)	C6—N2—C7—C12	49.1 (15)
Cl1—Hg1—N1—C5	80.8 (9)	Hg1—N2—C7—C12	−119.7 (10)
N2—Hg1—N1—C5	3.9 (6)	C5—N1—C1—C2	0.3 (15)
Cl2—Hg1—N1—C5	−98.1 (6)	Hg1—N1—C1—C2	−172.9 (8)
Cl2^i^—Hg1—N1—C5	169.1 (6)	C8—C7—C12—C11	1.6 (19)
N1—Hg1—N2—C6	0.1 (6)	N2—C7—C12—C11	175.3 (12)
Cl1—Hg1—N2—C6	−161.6 (6)	C7—C8—C9—C10	1.1 (18)
Cl2—Hg1—N2—C6	91.2 (6)	C13—C8—C9—C10	179.5 (11)
Cl2^i^—Hg1—N2—C6	−35.8 (10)	C7—C8—C9—C14	−177.9 (13)
N1—Hg1—N2—C7	169.7 (9)	C13—C8—C9—C14	0.5 (19)
Cl1—Hg1—N2—C7	8.0 (8)	N1—C1—C2—C3	−0.7 (17)
Cl2—Hg1—N2—C7	−99.2 (8)	N1—C5—C4—C3	−0.5 (14)
Cl2^i^—Hg1—N2—C7	133.8 (7)	C6—C5—C4—C3	−179.3 (8)
C1—N1—C5—C4	0.3 (14)	C1—C2—C3—C4	0.5 (16)
Hg1—N1—C5—C4	173.8 (7)	C5—C4—C3—C2	0.1 (15)
C1—N1—C5—C6	179.1 (8)	C7—C12—C11—C10	−1 (2)
Hg1—N1—C5—C6	−7.5 (11)	C12—C11—C10—C9	1 (2)
C7—N2—C6—C5	−174.6 (8)	C8—C9—C10—C11	−1 (2)
Hg1—N2—C6—C5	−3.9 (11)	C14—C9—C10—C11	178.3 (15)
N1—C5—C6—N2	7.8 (13)		

Symmetry code: (i) −*x*+1/2, −*y*+3/2, −*z*+2.

### Hydrogen-bond geometry (Å, º)

**Table:**

*D*—H···*A*	*D*—H	H···*A*	*D*···*A*	*D*—H···*A*
C1—H1···Cl2^i^	0.93	2.65	3.400 (11)	138
C4—H4···Cl2^ii^	0.93	2.67	3.552 (11)	158
C14—H14*C*···Cl1^iii^	0.96	2.76	3.685 (14)	162

Symmetry codes: (i) −*x*+1/2, −*y*+3/2, −*z*+2; (ii) −*x*+1, *y*, −*z*+3/2; (iii) −*x*+1, −*y*+1, −*z*+2.
